# Contrast Sensitivity Deficits and Its Structural Correlates in Fuchs Uveitis Syndrome

**DOI:** 10.3389/fmed.2022.850435

**Published:** 2022-05-19

**Authors:** Fang-Yue Zhou, Yi-Sha Li, Xingneng Guo, Xiutong Shi, Ke Wu, Jing-Wei Zheng, Xia-Xin Li, Jiaqing Wu, Ruru Liu, Ma-Li Dai, Xiu-Feng Huang, Fang Hou, Dan Lin, Yu-Qin Wang

**Affiliations:** ^1^School of Ophthalmology & Optometry and Eye Hospital, Wenzhou Medical University, Wenzhou, China; ^2^School of Optometry, Tianjin Vocational Institute, Tianjin, China; ^3^The Second Affiliated Hospital, Wenzhou Medical University, Wenzhou, China; ^4^Wenzhou Medical University-Monash BDI Alliance in Clinical and Experimental Biomedicine, Wenzhou Medical University, Wenzhou, China

**Keywords:** Fuchs uveitis syndrome, contrast sensitivity, qCSF, grading of haze, optical coherence tomography angiography

## Abstract

**Purpose:**

To investigate the deficits in contrast sensitivity in patients with Fuchs uveitis syndrome (FUS) and to explore the potential relationship between contrast sensitivity and ocular structure.

**Methods:**

In this prospective study, 25 patients with FUS and 30 healthy volunteers were recruited. Eyes were divided into three groups: FUS-affected eyes (AE), fellow eyes (FE), and healthy eyes. The contrast sensitivity function (CSF) of all participants was evaluated using the quick CSF (qCSF) method. Fundus photographs were collected for the analysis of refractive media, and vascular density (VD) was assessed using optical coherence tomography angiography (OCTA). Data were analyzed and compared using the generalized estimating equation (GEE).

**Results:**

The CSF of AE was significantly lower than that of FE and controls, while no significant difference was observed between FE and controls. Contrast sensitivity was negatively correlated with the grade of haze. No significant correlation was found between visual function and VDs in FUS eyes.

**Conclusions:**

We found that the CSF of FUS-affected eyes was significantly reduced, and the visual impairment was predominantly caused by the refractive media turbidity.

## Introduction

Fuchs uveitis syndrome (FUS) is a chronic unilateral uveitis. It is the second most common type of non-infectious uveitis and accounts for 1–20% of all uveitis cases (1). Patients with FUS are typically between 17 and 50 years of age (2, 3), which means the impact of FUS on the visual functions can dramatically influence the long-term life quality of the patients because it happens at their most productive working ages. The main clinical feature of FUS is mild anterior uveitis, such as mild anterior chamber reaction and stellate keratic precipitates (3, 4). The symptomatic exacerbation of FUS could be treated with a short course of topical corticosteroids (5). If complications, such as cataracts and vitreous opacities, do not occur or have been successfully treated by surgery, the visual acuity of patients with FUS can remain stable (6–8).

Patients with FUS often complain of poor visual quality despite normal visual acuity test results (4, 9), due to the changes in ocular structure led by chronic inflammation in FUS eyes. FUS is a type of anterior uveitis, and refractive media turbidity, such as cataract and vitreous opacity, is commonly observed (4, 9). In addition, numerous studies have demonstrated that the vasculature of the retina is also affected, including the lower vascular density of macular and peripheral vascular leakage (10–15). It is not clear whether and how the ocular changes in FUS affect visual function. We hypothesize that the structural changes in refractive media and retinal vasculature could lead to visual impairments in FUS eyes.

Visual acuity, which records vision in high-contrast conditions, is widely used to assess visual impairment in clinical practice (16). However, studies have shown that the sensitivity of visual acuity is not high enough to detect small changes (17, 18). Therefore, a more sensitive tool is needed to appropriately assess visual function in FUS. Contrast sensitivity function (CSF) can comprehensively assess visual acuity at a range of spatial (17), but conventional laboratory CSF tests are time-consuming (19), and clinical charts, such as the Pelli-Robson chart, could only measure contrast sensitivity at an individual spatial frequency (20). The Bayesian adaptive quick CSF (qCSF) method is a computerized test designed to assess visual performance in a comprehensive yet efficient manner (19). Previous studies have proven that this metric's reproducibility and accuracy are superior to those of conventional assays (21). The qCSF has been used to assess visual impairment in multiple sclerosis (22), age-related macular degeneration (23), retinal vein occlusion (24), and various other ophthalmic diseases. Therefore, the qCSF may provide a suitable tool for clinical functional assessment in patients with FUS.

Either in previous reports (4, 25–27) or our clinical observations, few significant organic retinal changes were observed in FUS eyes *via* fundus photo and optical coherence tomography. However, many studies have demonstrated that the retinal vasculature of FUS is affected. Using optical coherence tomography angiography (OCTA), Aksoy et al. reported that the foveal vascular density (VD) of FUS eyes was significantly lower than the fellow eyes and controls (12). Summarizing previous studies related to fundus fluorescein angiography (28–30), Bouchenaki et al. concluded that retinal vascular leaking might be associated with FUS (15). OCTA and FFA can both visualize retinal vasculature. No significant leakage of vasculature was found in the macula in FUS eyes (4, 15, 28, 30). While, OCTA visualizes static retinal vasculature, and showed a decrease in macular VD in FUS eyes (12). In addition, OCTA is non-invasive, which means it is safer in clinics. There have been other studies on diseases, such as diabetes (31) and age-related macular degeneration (32), found that the reduced contrast sensitivity was associated with VD. So we chose OCTA to explore the correlation between retinal structure and contrast sensitivity.

In this study, we aimed to explore the contrast sensitivity deficits and the potential relationship between contrast sensitivity and ocular structure of FUS eyes. The contrast sensitivities of patients with FUS and healthy participants were measured by qCSF. The structural changes were investigated in two parts: the condition of refractive media estimated by fundus photographs and the VD analyzed using OCTA. The functional and structural measurements were then compared between the FUS and healthy eyes, and their relationships were explored.

## Materials and Methods

### Study Subjects

As a prospective study, we focused on the patients with FUS who were seen in the clinic of the Eye Hospital of Wenzhou Medical University between January 2020 and December 2021. The study followed the Helsinki Principles and was approved by the ethics committee of The Eye Hospital of Wenzhou Medical University. Informed consent was obtained from all participants. The best-corrected visual acuity (BCVA) was expressed by a standard logarithmic visual acuity chart in logMAR (33). The participants' medical records were collected, de-identified, and analyzed.

According to the diagnostic criteria proposed by Yang (34) and SUN (3), we determined that the FUS diagnosis should meet the typical clinical findings seen by slit-lamp biomicroscopy: mild uveitis (mild anterior chamber reaction and stellate keratic precipitates), diffuse parenchymal atrophy of iris, and the lack of posterior synechiae. Our exclusion criteria were as follows: (1) spherical equivalent value > −6.0 diopter or +3.0 diopter of refractive error and/or axial length > 26 mm; (2) binocular axial length difference ≥1 mm; (3) co-existing eye diseases other than FUS; (4) ocular manifestations of glaucoma, such as glaucomatous optic disc changes or intraocular pressure ≥ 21 mmHg; and (5) intraocular surgery history in the past 3 months. All patients were diagnosed and examined by the same uveitis specialist (YQW). The involved controls with no significant ocular or systemic disease were matched for age, spherical equivalent value, and axial length to patients with FUS who participated.

In this study, 25 patients with FUS and 30 healthy participants were recruited. Both eyes of all participants were examined. Since five patients had bilateral FUS, totally there were 30 affected eyes (AE), 20 fellow eyes (FE), and 60 healthy eyes.

To evaluate the resolution of fundus images, two ophthalmologists (FYZ and DL) independently graded fundus photographs according to the 9-step photographic haze grading scale (35). If the results were different, a senior retinal specialist (YQW) provided the final grading.

### qCSF Measurements

The qCSF device (Manifold Contrast Vision Meter, Adaptive Sensory Technology, San Diego, California, USA) was used to obtain parameters of contrast sensitivity (36). Participants were asked to report the digits presented on the display of Manifold. The examiner recorded the responses as “correct,” “incorrect,” or “no response” using a handheld tablet. With the best correction by glasses, each eye was tested separately in 25 trials. The following CSF paraments were calculated: (I) the area under the log contrast sensitivity function (AULCSF), reflecting the summary metric of CSF precisely (37); (II) CSF acuity, the spatial frequency at which contrast sensitivity was 100%; and (III) contrast sensitivity thresholds at 1, 1.5, 3, 12, and 18 cycle per degree (cpd).

### OCTA Measurements

All OCTA images were obtained using AngioVue software (Version 2017.1.0.151) of the RTVue XR Avanti (Optovue, Inc., Fremont, CA). Optovue density function software was used for further analysis. Vascular density (VD) is defined by the percentage of the image that is occupied by blood vessels. The sets of scans were acquired according to the AngioVue macular cube (3 mm × 3 mm) protocol. Only the images with signal strength index ≥6 and without motion artifacts or segmentation errors were included for analysis.

The retina macular area was automatically divided into superficial capillary plexus (SCP) and deep capillary plexus (DCP). The SCP is defined as occurring between the inner limiting membrane and 10 μm above the inner plexiform layer. The DCP is defined as occurring between 10 μm above the inner plexiform layer and 10 μm beneath the outer plexiform layer. Based on the contour of the Early Treatment Diabetic Retinopathy Study (ETDRS), the macular area was divided into five areas: the fovea, upper side, lower side, nasal side, and temporal side. The software was used to measure VD in each part of the macular area.

### Statistical Analysis

For statistical analysis, SPSS 18 (Windows version 18; SPSS, Inc.) was used. Values are presented as mean ± standard deviation (SD). A chi-square test analysis was used for categorical variable groups. Data distribution was analyzed using Shapiro–Wilk's test. As the data for demographic and clinical characteristics were not normal, the Kruskal–Wallis statistic was used to detect significant differences. To correct for the relatedness in the data between two eyes, the generalized estimating equation (GEE) was applied with a within-subject factor to analyze the data of the patients with FUS and normal subjects. FE and AE were compared in univariable analyses to evaluate the influence of ocular structure on CSF. The value of *p* < 0.05 was considered statistically significant. GraphPad Prism 7.0 was used to plot charts.

## Results

The demographic and clinical characteristics of all participants are listed in Table 1. No significant differences were found between the three groups in terms of gender (*p* = 0.729), age (*p* = 0.968), axial length (*p* = 0.495), and spherical equivalent (*p* = 0.841). Despite similar numbers, BCVA was found to be statistically different between the AE and control groups (*p* < 0.001), while no significant difference was observed between FE and control groups (*p* = 0.140). Haze grading was significantly different between AE and control groups (*p* < 0.001), while no significant difference was observed between FE and control groups (*p* > 0.999).

**Table 1:** Demographic and clinical characteristics of study participants.

		**FUS patients (*n* = 25)**	**Controls (*n* = 30)**	***P***
**Laterality**	Unilateral	20		
	Bilateral	5		
**Gender**	Female	13	17	0.729^*^
	Male	12	13	
**Age**	AE	41.2 ± 8.9	41.4 ± 10.5	0.968^†^
	FE	41.8 ± 10.6		
**Axial length (mm)**	AE	23.46 ± 0.79	23.74 ± 0.88	0.495^†^
	FE	23.60 ± 0.92		
**SE (Diopters)**	AE	−0.53 ± 1.37	−0.59 ± 1.23	0.814^†^
	FE	−1.04 ± 1.72		
**BCVA (log MAR)**	AE	0.05 ± 0.09	−0.04 ± 0.07	**<0.001** ^†^
	FE	−0.01 ± 0.06		0.140^†^
**Grading of Haze**	AE	1.3 ± 1.2	0.1 ± 0.3	**<0.001** ^†^
	FE	0.2 ± 0.4		>0.999^†^

*Values are depicted as mean ± standard deviation (SD)*.

*SE, Spherical equivalent; BCVA, best-corrected vision acuity; AE, Fuchs uveitis syndrome (FUS)-affected eyes of patients; FE, fellow eyes of patients; p, the value of p*.

* *Chi-square test; Kruskal–Wallis statistic*.

† *Bold values indicate a statistical significance of p < 0.05*.

### Contrast Sensitivity of FUS Eyes Is Reduced

**Figure 1:**
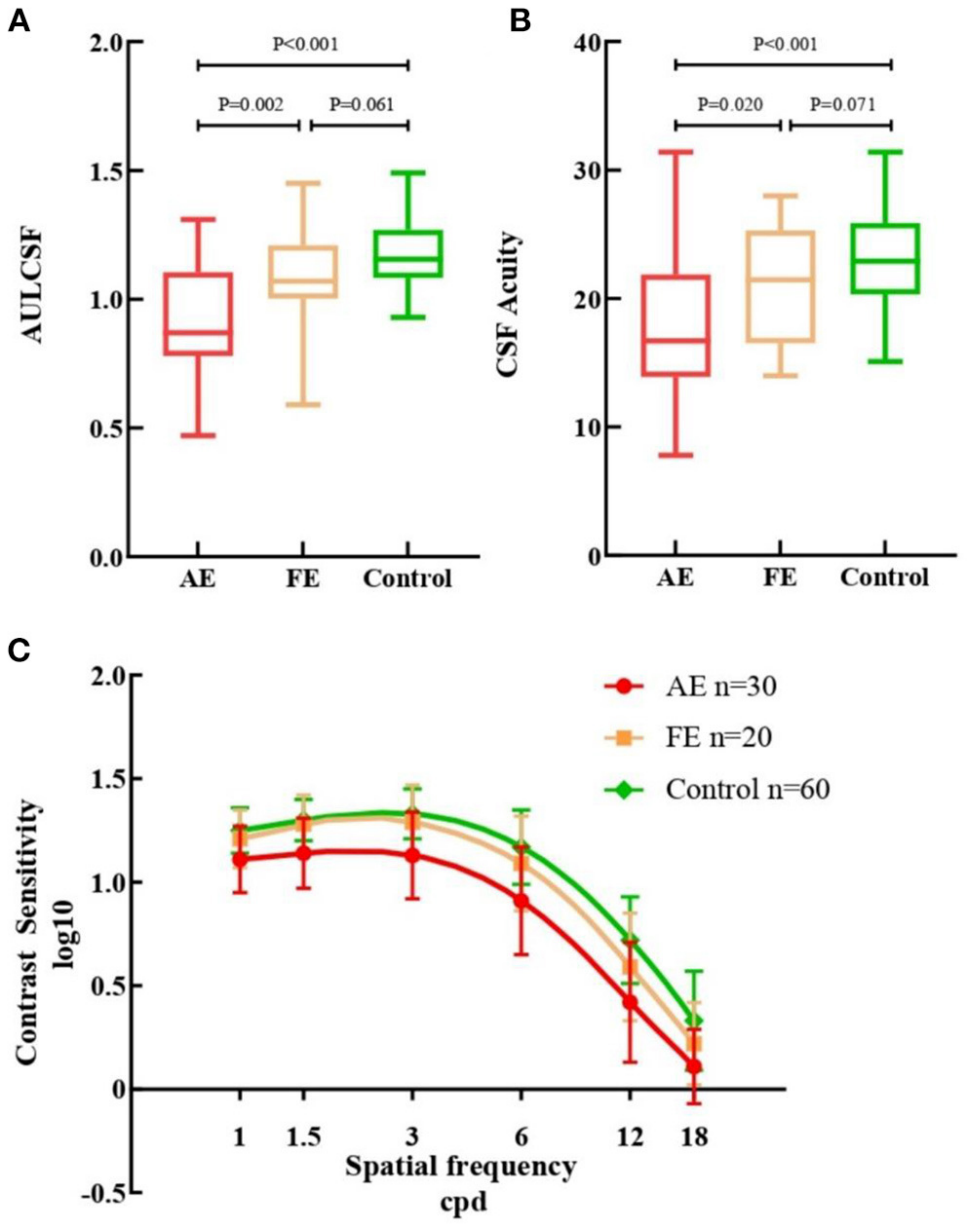
Measurements from quick contrast sensitivity function testing. **(A)** Box plots of AULCSF for the three groups. **(B)** Box plots of CSF Acuity for the three groups. **(C)** Contrast sensitivity of all cpd in AE (red), FE (yellow) and controls (green). AULCSF, the area under log contrast sensitivity function; CSF acuity, the spatial frequency at which contrast sensitivity was 100%; AE, FUS-affected eyes of patients; FE, fellow eyes of patients. Error bars represent ± standard deviation (SD); cpd, cycle per degree.

The difference among the three groups was tested with GEE analyses, correcting for binocular relatedness. Results showed that there was a consistent stepwise difference among the three studied groups in terms of QCF parameters (Figure 1). The AULCSF value of AE (0.91 ± 0.21) was lower than that of both FE [1.09 ± 0.20, β −0.176, 95% *CI* (−0.289, −0.062), *p* = 0.002] and control [1.18 ± 0.13, β −0.266, 95% *CI* (−0.354, −0.177), *p* **<** 0.001] groups. The CSF acuity value for the AE group (17.57 ± 5.44) was similar to that of the FE group [21.06 ± 4.77, β −3.487, 95% *CI* (−6.426, −0.548), *p* = 0.020] and the control group [23.19 ± 3.90, β −5.617, 95% *CI* (−8.066, −3.167), *p* **<** 0.001]. No statistical differences were observed in AULCSF [β −0.090, 95% *CI* (−0.189, 0.009), *p* = 0.061] or CSF acuity [β −2.130, 95% *CI* (−4.549, 0.289), *p* = 0.071] between FE and control groups. Contrast sensitivity reductions were seen in AE at all spatial frequencies when compared with FE and control groups, especially at low and intermediate spatial frequencies. There were almost no statistical differences between the FE and control groups.

### Outcomes of OCTA

As shown in Table 2, the three groups' vessel densities in the macular superficial and deep capillary plexus were compared. In the assessment of SCP, VD was significantly lower in the full image for AE (42.35 ± 4.75) compared with FE [45.63 ± 4.29, β −3.280, 95% *CI* (−5.399, −1.161), *p* = 0.002] and control [47.08 ± 2.54, β −4.704, 95% *CI* (−6.607, −2.801), *p* < 0.001] groups. In the assessment of DCP, VD in the full image for AE (47.63 ± 4.40) was also lower than FE [50.86 ± 2.90, β −3.227, 95% *CI* (−4.817, −1.638), *p* < 0.001] and control groups [50.50 ± 2.85, β −2.858, 95% *CI* (−4.583, −1.132), *p* = 0.001]. Though the similar results that VD for AE was lower than FE and control groups were showed in the temporal, superior, nasal, and inferior regions, no significant differences in foveal VD were found among three groups. The differences between FE and control groups were not statistically significant.

**Table 2:** Comparison of vessel densities in the macular superficial and deep capillary plexus among groups.

	**AE**	**FE**	**Controls**	**AE vs. FE**				**AE vs. controls**				**FE vs. controls**			
**Superficial VD%**				**β**	**95%CI**	**Wald χ^2^**	***P***	**β**	**95%CI**	**Wald χ^2^**	***P***	**β**	**95%CI**	**Wald χ^2^**	***P***
Whole image	42.35 ± 4.75	45.63 ± 4.29	47.08 ± 2.54	−3.280	−5.399, −1.161	9.208	**0.002**	−4.704	−6.607, −2.801	23.478	**<0.001**	−1.424	−3.388, 0.54	2.019	0.155
Fovea	14.36 ± 5.33	14.26 ± 4.87	15.77 ± 5.17	0.103	−2.456, 2.662	0.006	0.937	−1.314	−4.219, 1.591	0.786	0.375	−1.417	−4.123, 1.289	1.054	0.305
Temporal	44.04 ± 4.07	46.74 ± 4.43	48.39 ± 3.42	−2.697	−4.661, −0.732	7.234	**0.007**	−4.355	−6.064, −2.646	24.949	**<0.001**	−1.659	−3.757, 0.440	2.398	0.121
Superior	45.94 ± 5.93	49.54 ± 5.18	51.25 ± 2.89	−3.605	−5.932, −1.278	9.219	**0.002**	−5.223	−7.562, −2.884	19.153	**<0.001**	−1.618	−3.999, 0.763	1.775	0.183
Nasal	44.02 ± 6.05	47.45 ± 5.22	49.18 ± 2.68	−3.426	−6.332, −0.519	5.337	**0.021**	−5.135	−7.550, −2.721	17.377	**<0.001**	−1.710	−4.054, 0.634	2.044	0.153
Inferior	46.46 ± 5.47	50.30 ± 5.53	51.65 ± 3.34	−3.833	−6.402, −1.263	8.548	**0.003**	−5.245	−7.426, −3.063	22.208	**<0.001**	−1.412	−3.948, 1.124	1.191	0.275
**Deep VD%**															
Whole image	47.63 ± 4.40	50.86 ± 2.90	50.50 ± 2.85	−3.227	−4.817, −1.638	15.830	**<0.001**	−2.858	−4.583, −1.132	10.532	**0.001**	0.370	−1.134, 1.873	0.232	0.630
Fovea	26.67 ± 6.97	27.48 ± 6.66	29.21 ± 6.86	−0.812	−3.987, −2.362	0.251	0.616	−2.546	−6.279, 1.187	1.787	0.181	−1.734	−5.419, 1.952	0.850	0.357
Temporal	51.11 ± 4.44	53.75 ± 3.14	53.06 ± 2.96	−2.639	−4.378, −0.899	8.842	**0.003**	−1.949	−3.760, −0.138	4.447	**0.035**	0.690	−0.947, 2.326	0.682	0.409
Superior	50.19 ± 5.07	53.76 ± 2.87	52.93 ± 3.36	−3.575	−5.260, −1.889	17.278	**<0.001**	−2.744	−4.763, −0.724	7.092	**0.008**	0.831	−0.741, 2.403	1.073	0.300
Nasal	51.35 ± 4.15	54.34 ± 2.73	53.61 ± 3.09	−2.990	−4.710, −1.269	11.592	**0.001**	−2.258	−3.980, −0.535	6.600	**0.010**	0.732	−0.780, 2.243	0.901	0.343
Inferior	49.50 ± 5.33	53.23 ± 4.07	52.11 ± 3.51	−3.729	−5.979, −1.480	10.556	**0.001**	−2.608	−4.692, −0.524	6.015	**0.014**	1.122	−0.925, 3.168	1.154	0.283

*Values are depicted as mean ± SD*.

*GEE analyses, AE, FUS-affected eyes of patients; FE, fellow eyes of patients; VD, vascular density; GEE, generalized estimating equation; CI, confidence interval; β, regression coefficient; and p, the value of p*.

*Bold values indicate a statistical significance of p < 0.05*.

### AULCSF Is Correlated With Haze Grading

The correlation analysis between contrast sensitivity and refractive media, and the correlation analysis between contrast sensitivity and retinal vasculature were performed in patients with FUS, and the outcomes are listed in Table 3. No significant correlation was found between BCVA and structures. AULCSF exhibited a statistical correlation with haze grading in AE [β −0.084, 95% *CI* (−0.136, −0.031), *p* = 0.002] and FE [β −0.387, 95% *CI* (−0.590, −0.184), *p* < 0.001], while no significant correlations were observed between AULCSF and VDs for the whole image of SCP and DCP (*p* > 0.05). About CSF acuity, negative correlations with the grading of haze were observed in AE [β −1.561, 95% *CI* (−3.073, −0.049), *p* = 0.043] and FE [β −4.816, 95% CI (−9.092, −0.539), *p* = 0.027], while no statistical correlation was observed between CSF acuity and VDs (*p* > 0.05).

**Table 3:** Visual function–ocular structure correlation of FUS eyes.

	**BCVA**								**AULCSF**								**CSF acuity**							
	**AE**				**FE**				**AE**				**FE**				**AE**				**FE**			
	**β**	**(95%CI)**	**Wald χ^2^**	***P***	**β**	**(95%CI)**	**Wald χ^2^**	***P***	**β**	**(95%CI)**	**Wald χ^2^**	***P***	**β**	**(95%CI)**	**Wald χ^2^**	***P***	**β**	**(95%CI)**	**Wald χ^2^**	***P***	**β**	**(95%CI)**	**Wald χ^2^**	***P***
Grading of Haze	0.011	(−0.012, 0.034)	0.841	0.359	0.051	(−0.008, 0.110)	2.903	0.088	−0.084	(−0.136, −0.031)	9.847	**0.002**	−0.387	(−0.590, −0.184)	13.999	**<0.001**	−1.561	(−3.073, −0.049)	4.094	**0.043**	−4.816	(−9.092, −0.539)	4.871	**0.027**
SCP	−0.002	(−0.008, 0.004)	0.455	0.500	−0.003	(−0.008, 0.001)	2.058	0.151	0.011	(−0.002, 0.024)	2.870	0.090	0.019	(0.000, 0.038)	3.806	0.051	0.253	(−0.087, 0.594)	2.123	0.145	0.449	(0.029, 0.870)	4.381	0.036
DCP	−0.003	(−0.010, 0.004)	0.576	0.448	−0.004	(−0.011, 0.002)	1.735	0.188	0.009	(−0.009, 0.027)	0.920	0.337	0.016	(−0.014, 0.046)	1.081	0.298	0.290	(−0.088, 0.667)	2.261	0.133	0.014	(−0.617, 0.644)	0.002	0.966

*GEE analyses, AE, FUS-affected eyes of patients; FE, fellow eyes of patients; SCP, superficial capillary plexus; DCP, deep capillary plexus. GEE, generalized estimating equation; CI, confidence interval; β, regression coefficient; and p, the value of p*.

*Bold values indicate a statistical significance of p < 0.05*.

## Discussion

In this study, we evaluated the contrast sensitivity and ocular structure of patients with FUS to elucidate whether and how the structural changes in the refractive media and retinal vasculature impaired visual function.

We used qCSF to evaluate the contrast sensitivity of all participants. Compared with the fellow eyes and controls, the AULCSF of the FUS eyes was reduced, and the contrast sensitivity values of the FUS eyes at all spatial frequencies were also inferior (Figure 1). These analyses suggest that the CSF of the FUS eyes was lower than that of the fellow eyes and controls.

Numerous factors can affect CSF, we speculated that in the ocular structures, CSF may be mainly affected by two factors: (1) refractive media, which interrupts the transmission of light so that the visual marker could not be projected clearly onto the retina. Patients with FUS are typically 17–50 years old, and the affected lenses manifest posterior subcapsular turbidity rather than total opacification analogous to age-related cataracts. It is inappropriate to evaluate the cloudy condition of the lens with the Lens Opacities Classification System III (38), and the vitreous turbidity degree could not be independently evaluated with our tools. Otherwise, turbidity could also be seen in other refractive media of the FUS eyes, including keratic precipitates of the corneal surface and cells in the anterior chamber due to chronic inflammation (39). The presence of keratic precipitates (30/30), posterior subcapsular turbidity (20/30), and vitreous opacity (27/30) could be observed in most enrolled FUS eyes. However, because the refractive media was clouded to various degrees, it was difficult to evaluate each refractive media condition separately. Therefore, we opted to use the scale for photographic grading of vitreous haze (35) as the measure for the opacity of all refractive media. (2) Retinal structure affects the process of converting optical signals to electrical signals, so that the visual marker could not be recognized by the brain. In the pretest, we did not find a statistically significant difference in the macula thickness of FUS eyes compared with that of healthy eyes. A similar finding has been reported previously (25–27). Therefore, we did not choose to study the effect of macular thickness on CSF. Whereas, the choroidal results do not have a direct effect on the photoelectric conversion process as the retina does, we therefore chose VD *via* OCTA to explore the retinal vascular structure of FUS eyes.

The grading of haze in FUS eyes is noticeably inferior to that of healthy eyes, and the correlation between haze and contrast sensitivity was significant for the three groups. The results indicate that refractive media turbidity could be one of the causes of the reduced contrast sensitivity in FUS. It has been demonstrated in previous studies that vitreous turbidity is an important factor affecting vision after cataract surgery (40, 41); this also shows that vision could be affected significantly by refractive media turbidity, even in the state of diminished turbidity (clear lens). These results suggest that contrast sensitivity values can be used as a functional endpoint in patients with FUS whose refractive media are turbid. Contrast sensitivity could also be used to monitor changes in the patient's visual function and act as an indicator of the timing for cataract or vitrectomy surgery. In the future, we will analyze the effects of one of the refractive media, such as the lens or vitreous body, on CSF. It could help the clinic pinpoint the extent of each segment affecting vision and improve the vision of patients with FUS in a more targeted way.

In our study, both BCVA and CSF acuity showed the visual resolution limit of a patient when seeing a target. CSF acuity but not BCVA was found to be negatively correlated with the haze grading. Many studies have demonstrated that CSF could reflect the correlation between subjective visual function and vision-guided activities in daily life (42–44). Wai et al. showed that eyes with macular disease and good VA have significantly reduced CSF compared with healthy control eyes (45), and they thought CSF may be able to explain some subjective visual complaints and patients' bad visual experiences while visual acuity may not. Shamsi et al. have even put forward the idea that the visual acuity and contrast sensitivity in the foveal region were dissociative (46). Our study has demonstrated the above-mentioned ideas. Our study suggests that the combined assessment of contrast sensitivity and BCVA could be more comprehensive for patients with FUS.

The VDs of affected eyes were lower in all regions except the fovea. Aksoy et al. reported similar results (12). However, for the foveal region, we did not identify any significant differences in VD between SCP and DCP, while Aksoy et al. reported that the VDs of affected eyes were lower in SCP but similar in DCP. These differences in results may arise from lower values of macular VD, which require a much larger sample size to reveal any differences, individual differences, and/or different statistical methods.

Several studies have demonstrated that retinal structure is closely related to contrast sensitivity (31, 45, 47). Wang found that the retinal inner layer is associated with reduced contrast sensitivity in retinal vein occlusion (47). Shamsi et al. found that 36% of the variance in the contrast sensitivity could be explained by the retinal structure in patients with glaucoma, age-related macular degeneration, and normal people (46). In our study, we identified structural changes in the fundus of the eyes of patients with FUS *via* VD, but no significant correlation was observed between VDs and contrast sensitivity. Although we have tried to correct for the mutual influence between VDs and the grading of haze, there is still a possibility that the effect of VD reduction was relatively small compared with that of the refractive media so it could not be observed. In the future, we may explore the contrast sensitivity in patients with clear refractive media, such as the patients who had cataract surgery and/or vitrectomy. There is also another possibility that the retinal region of VDs that leads to visual impairment in FUS does not match the 3 mm × 3 mm macular region that was focused on in our study. It was reported that the thickness of the ganglion cell layer plus the inner plexiform layer within the retinal region between 1 and 2 mm eccentricities was highly correlated with contrast sensitivity (46). Therefore, in our future research, we can explore the correlation between different regions and layers of the retina and contrast sensitivity. Furthermore, it is known that choroidal structure changes exist in FUS eyes. Alev et al. and Muhammet et al. both reported that the choroid vascularity index in FUS eyes is significantly lower than that in healthy eyes (10, 11). Numerous studies have reported choroidal thickness thinning in FUS eyes (25, 26, 48, 49). The relationship between contrast sensitivity and choroidal structure needs to be investigated more deeply.

Our study showed that the haze, VD, and contrast sensitivity of fellow eyes of patients with FUS were not significantly different from those of normal eyes in general, consistent with existing knowledge. This result may appease the anxiety of patients because no significant ocular structural changes were found in their unaffected eyes. However, it is possible that a subclinical state existed in the fellow eyes, but it was too tiny to be observed.

In conclusion, both contrast sensitivity and VD were reduced in FUS' eyes, and the outcomes of fellow eyes and healthy eyes were similar. In addition, we found that contrast sensitivity reduction was associated with the grading of haze but was not significantly correlated with VD. Our results indicate that the visual impairment in FUS eyes is predominantly caused by refractive media turbidity.

## Data Availability Statement

The raw data supporting the conclusions of this article will be made available by the authors, without undue reservation.

## Ethics Statement

The studies involving human participants were reviewed and approved by the Ethics Committee of the Eye Hospital of Wenzhou Medical University. The patients/participants provided their written informed consent to participate in this study.

## Author Contributions

F-YZ and Y-SL developed the research ideas and drafted the manuscript. F-YZ, Y-SL, XG, XS, KW, X-XL, JW, and RL collected all the data. F-YZ and J-WZ analyzed the data. M-LD, FH, DL, and Y-QW finalized the manuscript for submission. All authors made contributions to this article and approved the submitted version.

## Funding

This study was supported by Scientific Research project of Zhejiang Provincial Education Department of China (Y202045475), Zhejiang Province Natural Science Foundation of China (LY21H180004, LQ20H180006, and LQ21H180011), and the Key R&D Program of Zhejiang Province (2021C04019).

## Conflict of Interest

The authors declare that the research was conducted in the absence of any commercial or financial relationships that could be construed as a potential conflict of interest.

## Publisher's Note

All claims expressed in this article are solely those of the authors and do not necessarily represent those of their affiliated organizations, or those of the publisher, the editors and the reviewers. Any product that may be evaluated in this article, or claim that may be made by its manufacturer, is not guaranteed or endorsed by the publisher.
